# The novel prognostic nomograms for predicting cancer-specific survival and overall survival in mixed medullary and follicular cell carcinoma: A SEER-based study

**DOI:** 10.1007/s00432-023-05326-5

**Published:** 2023-09-13

**Authors:** Yonghao Li, Tiantian Guo, Xuefei Gao, Jing Liu

**Affiliations:** 1 The First Clinical Medical College of Shanxi Medical University, Taiyuan, 030001 Shanxi China; 2 Department of Thyroid Surgery, The First Hospital of Shanxi Medical University, 85 South Jiefang Road, Taiyuan, 030001 Shanxi China

**Keywords:** Cancer-specific survival (CSS), Mixed medullary and follicular cell carcinoma (MMFCC), Overall survival (OS), Prognosis nomograms, Risk stratification

## Abstract

**Background:**

The aim of this study was to evaluate independent predictors of prognosis in patients with mixed medullary and follicular cell carcinoma (MMFCC) and to establish the novel prognostic nomograms in the academic community for 3-, 5-, and 10 year CSS and OS in patients with MMFCC.

**Methods:**

Demographic information, clinicopathological characteristics, treatment information, and survival status information of 200 patients with MMFCC and 6615 patients with medullary thyroid carcinoma (MTC) from 2000 to 2020 in the SEER database were retrospectively analyzed. Independent predictors of prognosis in MMFCC patients were derived using univariate and multivariate Cox regression analyses after relevant comparisons based on pathologic typing. On this basis, we developed and validated clinical prognostic nomograms and risk-stratified the patient population.

**Results:**

In this study, the clinical information of 200 patients with MMFCC was compared with that of 5947 patients with MTC (NOS) and 668 patients with MTC with amyloid stroma, and there was a significant difference in the relevant variables among the three, with the CSS being 88.5%, 87.5%, and 90.9%, and the OS being 76.5%, 75.4%, and 83.8%. Univariate and multivariate Cox regression analyses yielded that age at diagnosis, presence of distant metastases, thyroidectomy scope, and lymph node dissection status were significantly correlated with the prognosis of patients (*P* < 0.05), and were independent predictors of CSS and OS for patients with MMFCC, and the Kaplan–Meier survival curves plotted by these factors demonstrated their predictive power for the prognosis of patients with MMFCC. The concordance index of the prognostic nomograms of CSS and OS established on this basis was 0.838 and 0.794, respectively, and the time-dependent area under curve, calibration curve, and decision curve analysis curve showed that the model had good discriminative ability, accuracy, and clinical applicability.

**Conclusions:**

In this study, we concluded that there are large differences between MMFCC and MTC in terms of demographic information, clinicopathological characteristics, treatment information, and survival status information, and we constructed the novel prognostic nomograms for 3-, 5-, and 10 year CSS and OS for patients with MMFCC with risk stratification, which will help clinicians to develop individualized protocols for their postoperative treatments and follow-ups.

## Introduction

Mixed subtype thyroid carcinoma (MSTC) refers to malignant tumors with two or more different types of cells mixed in the thyroid gland of the same patient. According to the different sources, it is divided into two categories: 1. single follicular cell source, homologous tumors with multiple differentiation, i.e., mixed tumors with different differentiated follicular epithelial cell types (Liu et al. 2017). For example, differentiated thyroid carcinoma (DTC) combined with low-differentiated thyroid carcinoma and undifferentiated thyroid carcinoma, etc. Diversity, hybridity, and complexity are its main features. 2. Common origin of follicular cells and parafollicular C cells, i.e., mixed medullary and follicular cell carcinoma (MMFCC), in which follicular cell carcinoma refers to tumors originated from follicular epithelial cells in general, and the two components of medullary thyroid carcinoma (MTC) and DTC are mostly mixed in MMFCC (Ueki et al. 2011).

In 1988, the World Health Organization established the name and diagnostic criteria of MMFCC for the first time, which was defined as a mixed carcinoma of follicular epithelial cell origin that showed both calcitonin immunopositive MTC and thyroglobulin immunopositive follicular epithelial cell-derived malignant tumors (Hedinger et al. 1988). However, due to the rarity of MMFCC in clinical practice, there are no relevant guidelines and consensus on the diagnosis and treatment of MMFCC in the academic community, and the current studies mostly focus on individual case reports, and there is a lack of large-scale studies on its clinicopathologic features and prognosis, and there is no development and validation of the prognostic nomogram of MMFCC in the academic community. In addition, Ion Negura et al. (Negura et al. 2023) concluded that compared with PTC, in MMFCC, the medullary component was more aggressive than the papillary one, but also more aggressive than MTC-only tumors. Other previous studies have also concluded that the prognosis of MSTC depends on the tumor components with worse prognosis (Thomas et al. 2021). However, it is worth noting that the prognosis difference between MMFCC and MTC is still controversial (Liu et al. 2017; Sandilos et al. 2023). The SEER (surveillance, epidemiology, and end results) database program is a database produced by the National Cancer Institute to provide data on cancer-related incidence, staging, treatment, and patient survival. The database contains information from 18 population-based tumor registries covering 28% of the regional population in the USA. The purpose of this study was to compare the clinical characteristics of patients with MMFCC and MTC in the SEER database and to analyze the survival and prognostic factors of patients with MMFCC in order to construct the novel prognostic nomograms for patients with MMFCC in the academic community and to evaluate and risk-stratify them. This prognostic nomograms can help clinicians to evaluate the prognosis of MMFCC patients and develop personalized treatment plans for them.

## Materials and methods

### Study population

**Fig. 1:**
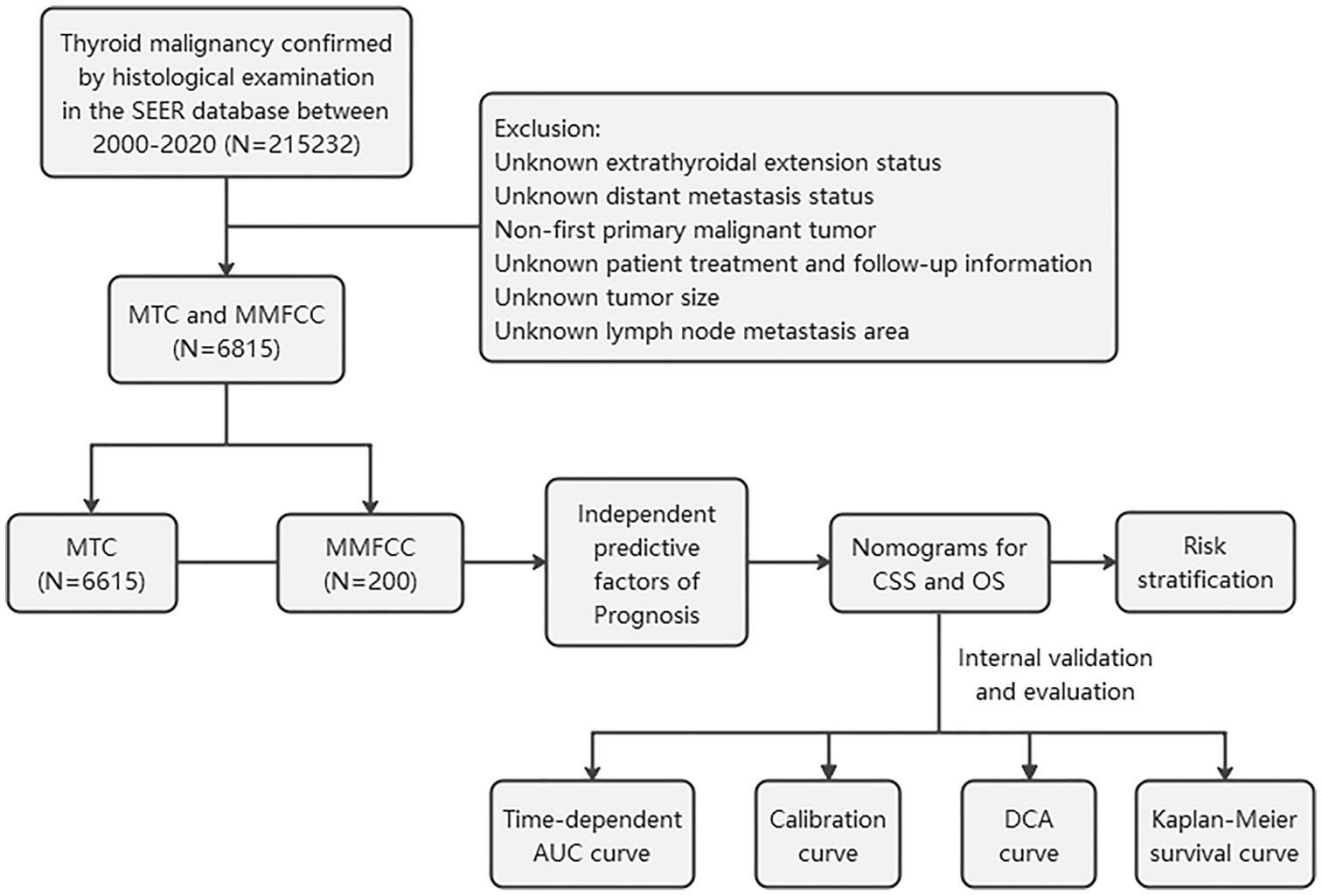
Flowchart of patients enrolled in this study

Patients included in this study were obtained from the SEER*STAT8.4.1 database, which retrospectively analyzed 215232 patients with thyroid malignancies diagnosed by pathology between 2000 and 2020. In order to compare the clinical characteristics and survival status of patients with MTC and MMFCC, we included 8874 patients with MTC (ICD-O-3: 8345/8510) and MMFCC (ICD-O-3: 8346/8347) patients into the study. MTC patients were divided into MTC (NOS) and MTC with amyloid stroma. MTC (NOS) means all MTC with unclear classification, that is, represents a broad group of MTC. The exclusion criteria were as follows: (1) tumor size, distant metastasis (DM) status, extraglandular invasion (ETE), and lymph node metastasis (LNM) region were unknown; (2) non-first primary tumor; (3) patient treatment and follow-up information was unknown. Because the SEER database does not release personally identifiable information, this study did not require the approval and consent of the Ethics Committee of the First Hospital of Shanxi Medical University. All authors have signed the author declaration form. A total of 6815 patients with MTC and MMFCC were finally enrolled in this study, and Fig. 1 shows the flowchart of the patient selection procedure in this study.

### Data selection

The endpoints of this study were cancer-specific survival (CSS) and overall survival (OS), with CSS defined as the time from the time of surgery to the date of death due to MMFCC or the date of the last follow-up, and OS defined as the time from the time of surgery to the date of death from any cause or the date of the last follow-up. A total of 14 variables were included in this study to identify independent predictors of CSS and OS in MMFCC patients. Demographic variables included gender, race, age at diagnosis, and marital status. MMFCC was categorized in the SEER database as mixed medullary-papillary thyroid carcinoma (MM-PTC) (ICD-O-3: 8347), and mixed medullary-follicular thyroid carcinoma (MM-FTC) (ICD-O-3: 8346). Clinicopathological characteristics included MMFCC subtype, tumor size, ETE status, LNM region, and the presence of DM. Treatment-related variables included thyroidectomy scope, lymph node dissection (LND) status, radiotherapy and chemotherapy status, and time from diagnosis of the first treatment. All methods were performed in accordance with the relevant guidelines of the SEER database.

### Statistical analysis

All statistical analyses in this study were performed using R software (Version 4.2.2, R Foundation for Statistic Computing, Austria) (http://www.r-project.org/) and SPSS (Version 25, IBM, USA). We grouped all patients according to the type of pathology (MTC (NOS), MTC with amyloid stroma, MM-PTC, and MM-FTC), followed by *χ*^2^ test, two independent samples *t* test, nonparametric rank-sum test, and one-way ANOVA test for MTC (NOS), MTC with amyloid stroma, and MMFCC as well as MM-PTC- and MM-FTC-related variables were compared. The optimal cut-off values for age and tumor size were evaluated by using the X-tile software in order to facilitate their classification and expressed as number and percentage. Independent predictors of CSS and OS in the retrospective data were evaluated using univariate Cox regression analysis, and variables with a *P* value < 0.1 in the univariate analysis were included in the multivariate Cox regression analysis, with a *P* value of < 0.05 considered statistically significant and identified as the final independent predictors of CSS and OS in patients with MMFCC, and the strength of the associations between each of the predictive categorical variables and survival was expressed as risk ratio (HR), while 95% confidence intervals (95% CI) were calculated. On this basis, the nomograms were plotted using R software, and the predictive performance of the prognostic nomograms was evaluated by calculating the C index (concordance index) and plotting the time-dependent area under the curve (AUC) curve, and the closer the value of the AUC is to 1, the better the predictive accuracy is. In addition, we evaluated the accuracy and clinical value of nomograms by plotting calibration curves and decision curve analysis (DCA) curves. The Kaplan–Meier survival curves were used to demonstrate the trends of CSS and OS in MMFCC patients under relevant variables. Based on the risk scores of each factor in the nomograms, we determined the cut-off value by calculating the Jordon index and divided it into two subgroups, low-risk and high-risk groups, and visualized the prognosis of MMFCC patients in this study by drawing the risk factor maps.

## Results

### Demographic information, clinicopathological features, and treatment follow-up information of MMFCC and MTC patients

A total of 200 patients with MMFCC were enrolled in this retrospective study, and their demographic and clinical characteristics were compared with those of 5947 patients with MTC (NOS) and 668 patients with MTC with amyloid stroma. As shown in Table 1, 12 variables including CSS, OS, survival time, tumor size, and DM status were significantly different (*P* < 0.05). In this study, the highest percentage of women in MTC (NOS) patients was 81.1%, and in terms of treatment status, 32.5% of MMFCC patients did not undergo LND, which is a high value compared to the MTC patient group; nevertheless, there was little difference between the two in terms of LNM region. At the same time, a higher percentage of MMFCC patients underwent postoperative radioisotopes and a lower percentage of beam radiation and chemotherapy compared to MTC patients. Although the MTC with amyloid stroma patient group had the highest incidence of DM (11.4%) and the shortest mean survival time of 59.44 (± 78.439) months, it had the highest CSS and OS (90.9%, 83.9%). There were 139 cases of MM-PTC and 61 cases of MM-FTC in the MMFCC patient population, and we compared the two and found that no significant difference was observed in any of the other variables, except for tumor size (*P* = 0.015). Nevertheless, the survival status of MM-PTC was better than that of MM-FTC, and the difference was more pronounced with a larger standard deviation, with a mean survival time of 91.27 (± 60.732) months for MM-PTC and 84.38 (± 65.693) months for MM-FTC, of which the median survival time was 73 months, with the shortest death occurring in only 2 months.

**Table 1:** Demographic information, clinicopathological features, and treatment follow-up information of MMFCC and MTC patients

Variables	Mixed medullary and follicular cell carcinoma (*N* = 200)	Medullary carcinoma (NOS) (*N* = 5947)	Medullary carcinoma with amyloid stroma (*N* = 668)	*P* value	Mixed medullary-papillary carcinoma (*N* = 139)	Mixed medullary-follicular carcinoma (*N* = 61)	*P* value
Age (years)				0.390			0.554
Mean(± sd)	55.36 (± 14.861)	54.51 (± 16.282)	52.91 (± 17.781)		54.94 (± 14.619)	56.30 (± 15.439)	
M (IQR)	57 (45,65.75)	54 (44,66)	55 (41.25,67)		55 (44,65)	59 (45.5,67)	
Sex				< 0.001			0.122
Female	118 (59.0%)	4823 (81.1%)	378 (56.9%)		77 (55.4%)	41 (67.2%)	
Male	82 (41.0%)	1124 (18.9%)	290 (43.4%)		62 (44.6%)	20 (32.8%)	
Marital status				0.227			0.659
Married	124 (62.0%)	3361 (56.5%)	379 (56.7%)		87 (62.6%)	37 (60.6%)	
Unmarried	66 (33.0%)	2351 (39.5%)	255 (38.2%)		44 (31.7%)	22 (36.1%)	
Unknown	10 (5.0%)	235 (4.0%)	34 (5.1%)		8 (5.8%)	2 (3.3%)	
Race				< 0.001			0.707
White	166 (83.0%)	4498 (75.6%)	544 (81.4%)		117 (84.2%)	49 (80.3%)	
Black	15 (7.5%)	1020 (17.2%)	53 (7.9%)		9 (6.5%)	6 (9.8%)	
Other*	19 (9.5%)	403 (6.8%)	59 (8.8%)		13 (9.4%)	6 (9.8%)	
Unknown	0 (0.0%)	26 (0.4%)	12 (1.8%)		0 (0.0%)	0 (0.0%)	
Tumor size (mm)				< 0.001			0.015
Mean (± sd)	23.32 (± 17.868)	30.90 (± 26.277)	25.56 (± 19.121)		21.94 (± 18.295)	26.46 (± 16.573)	
M (IQR)	18 (12,30.75)	25 (15,39)	22 (12,35)		16 (10,25)	21 (15,35)	
ETE				0.072			0.527
Absence	178 (89.0%)	5251 (88.3%)	570 (85.3%)		125 (89.9%)	53 (86.9%)	
Presence	22 (11.0%)	696 (11.7%)	98 (14.7%)		14 (10.1%)	8 (13.1%)	
LNM				< 0.001			0.408
No LNM	72 (36.0%)	3415 (57.4%)	246 (36.8%)		51 (36.7%)	21 (34.4%)	
CLNM	37 (18.5%)	1167 (19.6%)	173 (25.9%)		26 (18.7%)	11 (18.0%)	
LLNM	26 (13.0%)	661 (11.1%)	117 (17.5%)		21 (15.1%)	5 (8.2%)	
No LND	65 (32.5%)	704 (11.8%)	132 (19.8%)		41 (29.5%)	24 (39.3%)	
DM				< 0.001			0.441
Absence	189 (94.5%)	5633 (94.7%)	592 (88.6%)		133 (95.7%)	56 (91.8%)	
Presence	11 (5.5%)	314 (5.3%)	76 (11.4%)		6 (4.3%)	5 (8.2%)	
Thyroidectomy scope				< 0.001			0.358
None	3 (1.5%)	94 (1.6%)	20 (3.0%)		1 (0.7%)	2 (3.3%)	
Unilateral thyroidectomy and/or isthmectomy	18 (9.0%)	2276 (38.3%)	40 (6.0%)		12 (8.6%)	6 (9.8%)	
Total or subtotal thyroidectomy	179 (89.5%)	3577 (60.1%)	608 (91.0%)		126 (90.4%)	53 (86.9%)	
LND status				< 0.001			0.259
No LND	65 (32.5%)	704 (11.8%)	132 (19.8%)		41 (29.5%)	24 (39.3%)	
Yes, pLNs ≤ 4	94 (47.0%)	4507 (75.8%)	368 (55.1%)		66 (47.5%)	28 (45.9%)	
Yes, pLNs ≥ 5	41 (20.5%)	736 (12.4%)	168 (25.1%)		32 (23.0%)	9 (14.8%)	
Radiation				< 0.001			0.172
None/unknown	149 (74.5%)	4063 (68.3%)	584 (87.4%)		106 (76.3%)	43 (70.5%)	
Beam radiation	6 (3.0%)	1772 (29.8%)	65 (9.7%)		2 (1.4%)	4 (6.6%)	
Radioisotopes	45 (22.5%)	112 (1.9%)	19 (2.8%)		31 (22.3%)	14 (23.0%)	
Chemotherapy				< 0.001			0.759
No	196 (98.0%)	3586 (60.3%)	635 (95.1%)		137 (98.6%)	59 (96.7%)	
Yes	4 (2.0%)	2361 (39.7%)	33 (4.9%)		2 (1.4%)	2 (3.3%)	
Months from diagnosis to treatment				0.533			0.890
< One month	110 (55.0%)	3032 (51.0%)	342 (51.2%)		76 (54.7%)	34 (55.7%)	
≥ One month	90 (45.0%)	2915 (49.0%)	326 (48.8%)		63 (45.3%)	27 (44.3%)	
CSS	177 (88.5%)	5206 (87.5%)	607 (90.9%)	< 0.001	126 (90.7%)	51 (83.6%)	0.151
OS	153 (76.5%)	4486 (75.4%)	560 (83.8%)	< 0.001	110 (79.1%)	43 (70.5%)	0.207
Survival months				< 0.001			0.472
Mean (± sd)	89.17 (± 62.202)	115.41 (± 71.672)	59.44 (± 78.439)		91.27 (± 60.732)	84.38 (± 65.693)	
M (IQR)	82.5 (32.25,134)	111 (54,176)	21 (11,82.75)		84 (42,134)	73 (19,136.5)	

*ETE* extrathyroidal extension, *LNM* lymph node metastasis, *CLNM* central lymph node metastasis, *LLNM* lateral lymph node metastasis, *LND* lymph node dissection, *DM* distant metastasis, *pLNs* positive lymph nodes, *CSS* cancer-specific survival, *OS* overall survival

*Includes: American Indian, native Alaskan and Asian, Pacific Islander

### Construction and internal validation of clinical prognostic nomograms

**Fig. 2:**
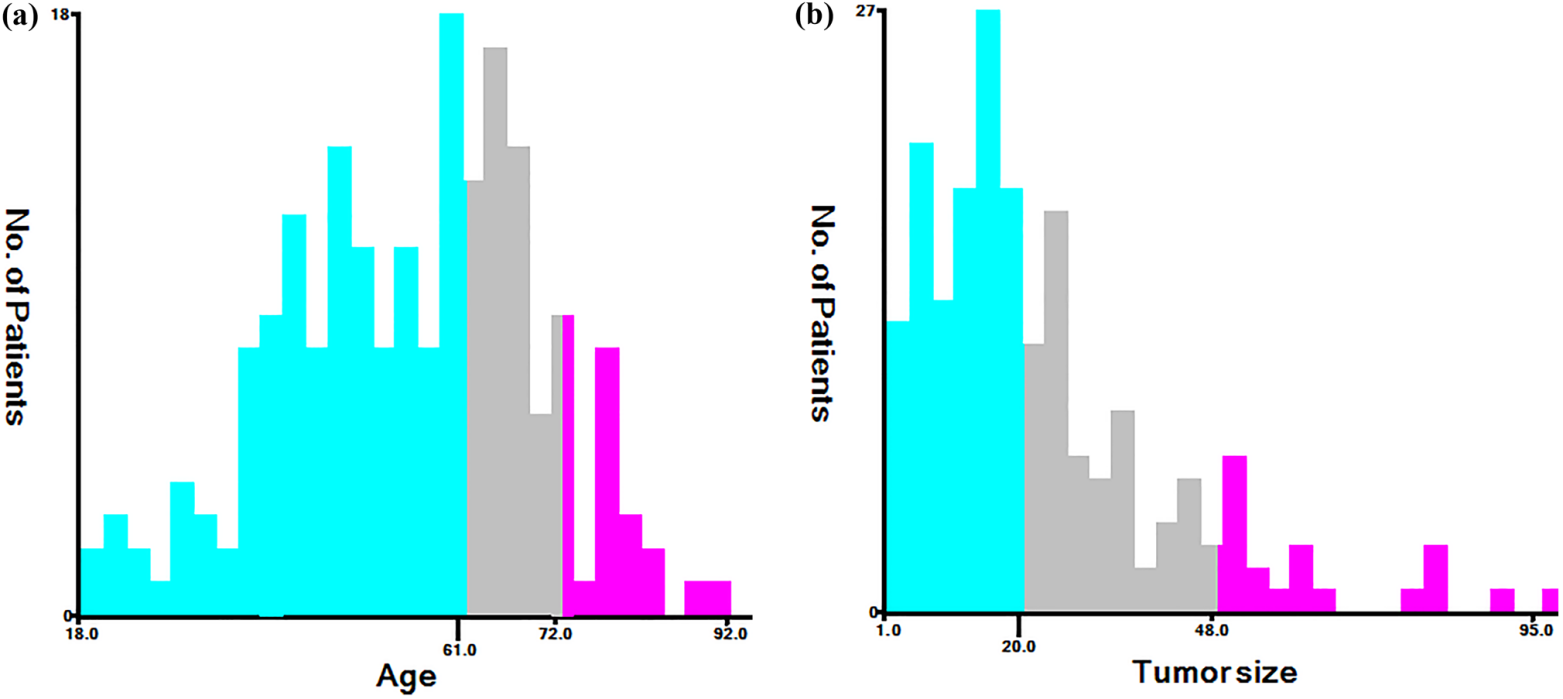
Optimal cut-off values for age (**a**) and tumor size (**b**) were identified by X-tile software analysis. According to cancer-specific survival, the optimal cut-off values of age at diagnosis were 61 and 72 years old, and the optimal cut-off values of tumor diameter were 20 mm and 48 mm

**Fig. 3:**
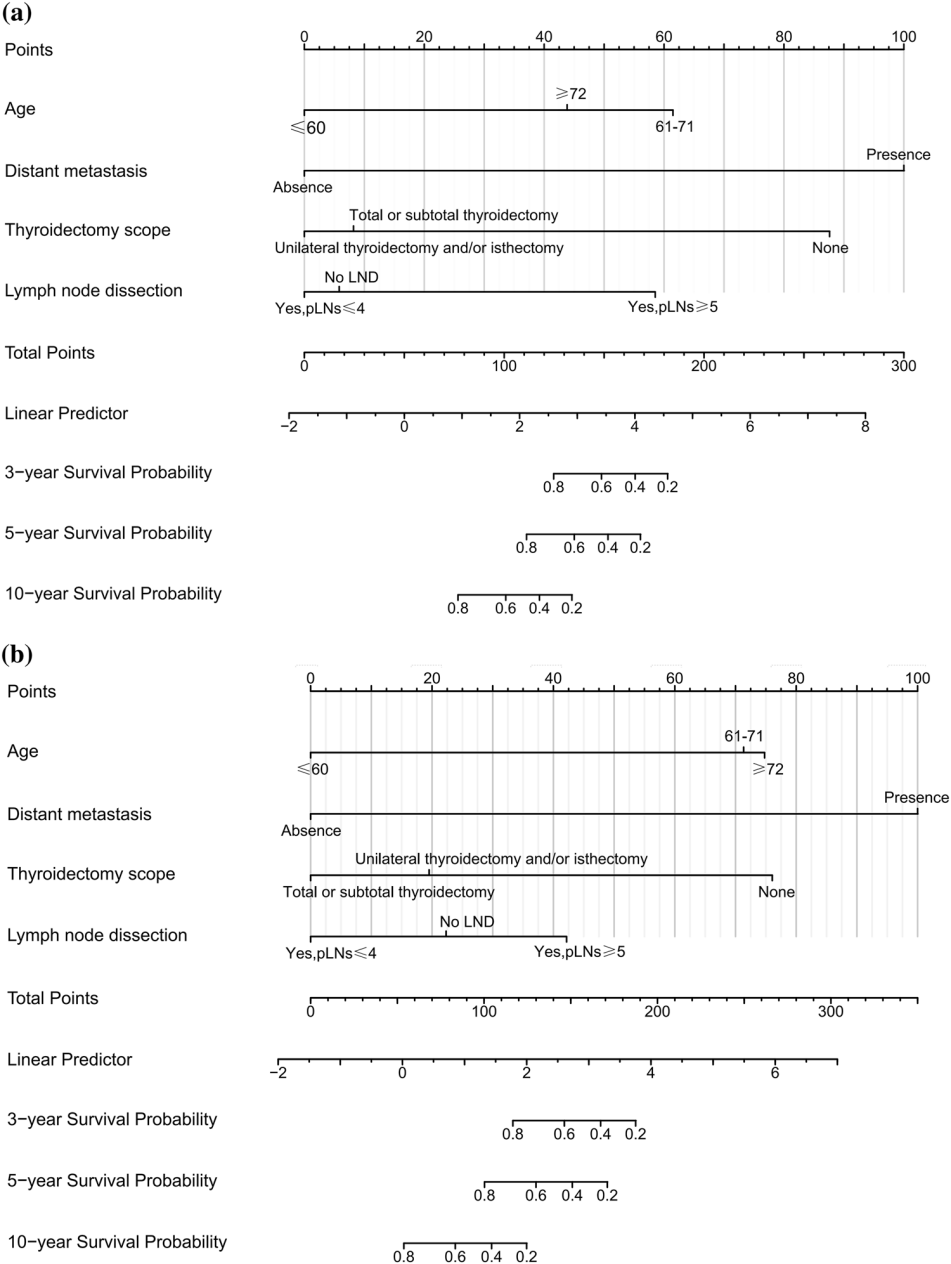
Nomograms to predict 3-, 5-, and 10 year cancer-specific survival (**a**) and overall survival (**b**) for MMFCC patients

The cut-off values of continuous variables for MMFCC patients were determined by X-tile software analysis, and the optimal cut-off values for age at diagnosis were 61 and 72 years old, and the optimal cut-off values for tumor size were 21 mm and 48 mm, as shown in Fig. 2a, b. Cox univariate and multivariate regression analyses according to this classification criterion yielded that age at diagnosis, the presence of DM, thyroidectomy scope, and LND status was significantly correlated with patient prognosis (*P* < 0.05) and were independent predictors of CSS and OS in MMFCC patients (Table 2, 3). Based on these findings, we established prognostic nomograms for CSS and OS in the MMFCC patient population (Fig. 3a, b).

**Table 2:** Univariate and multivariate cox regression analysis were used to analyze the cancer-specific survival

Variables	Univariate analysis		Multivariate analysis	
	HR (95% CI)	*P* value	HR (95% CI)	*P* value
Age (years)		0.030		
≤ 60	1		1	
61–71	2.865 (1.133–7.243)		8.396 (2.660–26.504)	< 0.001
≥ 72	3.402 (0.133–10.213)		4.401 (1.219–15.883)	0.024
Sex		0.080		
Female	1			
Male	2.091 (0.915–4.774)			
Marital status		0.674		
Married	1			
Unmarried	0.735 (0.285–1.896)			
Unknown	1.464 (0.334–6.412)			
Race		0.828		
White	1			
Black	0.564 (0.076–4.208)			
Other*	0.911 (0.213–3.900)			
Tumor size(mm)		0.001		
≤ 20	1			
21–47	2.963 (1.054–8.327)			
≥ 48	7.499 (2.598–21.646)			
ETE		0.004		
Absence	1			
Presence	4.045 (1.712–9.560)			
LNM		0.002		
No LNM	1			
CLNM	10.567 (2.315–48.231)			
LLNM	7.793 (1.417–42.858)			
No LND	3.970 (0.924–19.121)			
DM		< 0.001		
Absence	1		1	
Presence	26.839 (10.968–65.676)		30.973 (10.213–93.932)	< 0.001
Thyroidectomy scope		0.007		
None	1		1	
Unilateral thyroidectomy and/or isthmectomy	0.104 (0.017–0.641)		0.054 (0.005–0.591)	0.017
Total or subtotal thyroidectomy	0.042 (0.009–0.193)		0.071 (0.008–0.633)	0.018
LND status		0.024		
No LND	1		1	
Yes, pLNs ≤ 4	0.649 (0.218–1.934)		0.813 (0.235–2.815)	0.744
Yes, pLNs ≥ 5	2.548 (0.968–6.707)		6.100 (1.831–20.321)	0.003
Radiation		0.649		
None/unknown	1			
Beam radiation	1.016 (0.879–1.179)			
Radioisotopes	1.206 (0.819–1.775)			
Chemotherapy		0.001		
None	1			
Yes	18.998 (5.360, 67.322)			
Months from diagnosis to treatment		0.182		
< One month	1			
≥ One month	1.756 (0.769–4.007)			
Subtype		0.149		
MM-PTC	1			
MM-FTC	1.859 (0.815–4.242)			

*ETE* extrathyroidal extension, *LNM* lymph node metastasis, *CLNM* central lymph node metastasis, *LLNM* lateral lymph node metastasis, *LND* lymph node dissection, *DM* distant metastasis, *pLNs* positive lymph nodes, *MM-PTC* mixed medullary-papillary thyroid carcinoma, *MM-FTC* mixed medullary-follicular thyroid carcinoma

*Includes: American Indian, native Alaskan and Asian, Pacific Islander

**Table 3:** Univariate and multivariate cox regression analysis were used to analyze the overall survival

Variables	Univariate analysis		Multivariate analysis	
	HR (95% CI)	*P* value	HR (95% CI)	*P* value
Age (years)		< 0.001		
≤ 60	1		1	
61–71	4.426 (2.204–8.886)		7.273 (3.311–15.975)	< 0.001
≥ 72	7.256 (3.395–15.560)		7.910 (3.474–18.011)	< 0.001
Sex		0.297		
Female	1			
Male	1.362(0.765–2.422)			
Marital status		0.842		
Married	1			
Unmarried	1.016 (0.551–1.874)			
Unknown	0.676 (0.161–2.841)			
Race		0.764		
White	1			
Black	0.880(0.272–2.846)			
Other*	0.668(0.207–2.158)			
Tumor size (mm)		0.021		
≤ 20	1			
21–47	1.977 (1.047–3.736)			
≥ 48	2.791 (1.260–6.179)			
ETE		0.002		
Absence	1			
Presence	3.020 (1.590–5.739)			
LNM		0.043		
No LNM	1			
CLNM	2.559 (1.060–6.177)			
LLNM	2.510 (0.834–7.558)			
No LND	2.695 (1.240–5.857)			
DM		< 0.001		
Absence	1		1	
Presence	13.577 (6.507–28.330)		15.946 (6.745–37.698)	< 0.001
Thyroidectomy scope		0.005		
None	1		1	
Unilateral thyroidectomy and/or isthmectomy	0.175 (0.034–0.891)		0.228 (0.031–1.686)	0.147
Total or subtotal thyroidectomy	0.066 (0.015–0.284)		0.133 (0.020–0.892)	0.038
LND status		0.046		
No LND	1		1	
Yes, pLNs ≤ 4	0.446 (0.226–0.881)		0.534 (0.260–1.099)	0.089
Yes, pLNs ≥ 5	0.885 (0.431–1.817)		1.720 (0.762–3.844)	0.192
Radiation		< 0.001		
None/unknown	1			
Beam radiation	0.769 (0.689–0.859)			
Radioisotopes	0.969 (0.722–1.302)			
Chemotherapy		0.002		
None	1			
Yes	12.390 (3.673–41.802)			
Months from diagnosis to treatment		0.510		
< One month	1			
≥ One month	1.221(0.677–2.201)			
Subtype		0.174		
MM-PTC	1			
MM-FTC	1.516 (0.841–2.731)			

*ETE* extrathyroidal extension, *LNM* lymph node metastasis, *CLNM* central lymph node metastasis, *LLNM* lateral lymph node metastasis, *LND* lymph node dissection, *DM* distant metastasis, *pLNs* positive lymph nodes, *MM-PTC* mixed medullary-papillary thyroid carcinoma, *MM-FTC* mixed medullary-follicular thyroid carcinoma

*Includes: American Indian, native Alaskan and Asian, Pacific Islander

**Fig. 4:**
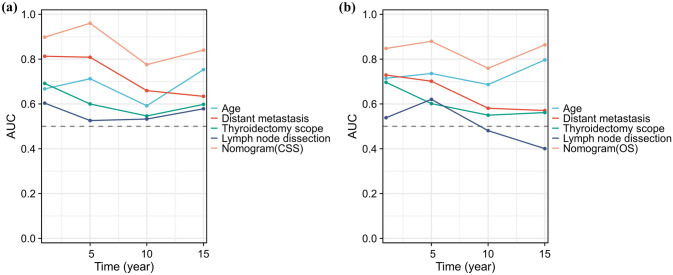
Each independent predictive factor with the established nomogram in the one time-dependent AUC curve: **a** nomogram (CSS); **b** nomogram (OS)

**Fig. 5.**
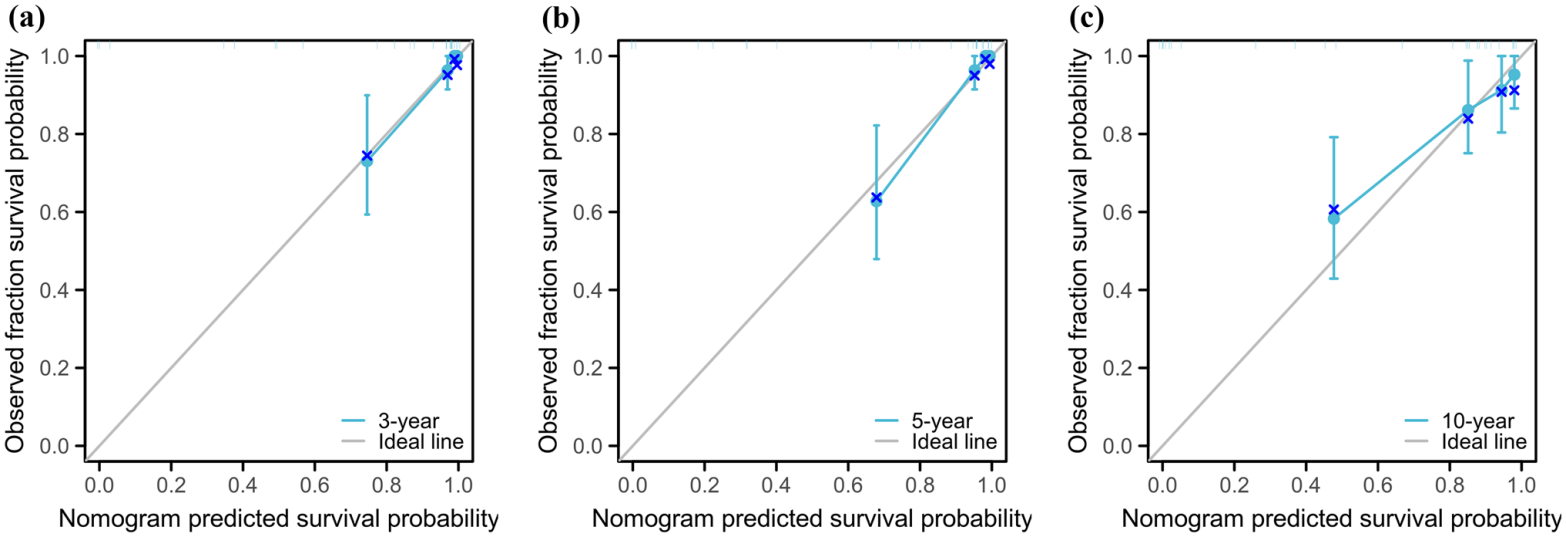
3 year (**a**), 5 year (**b**), and 10 year (**c**) cancer-specific survival nomogram internal verification curves

**Fig. 6.**
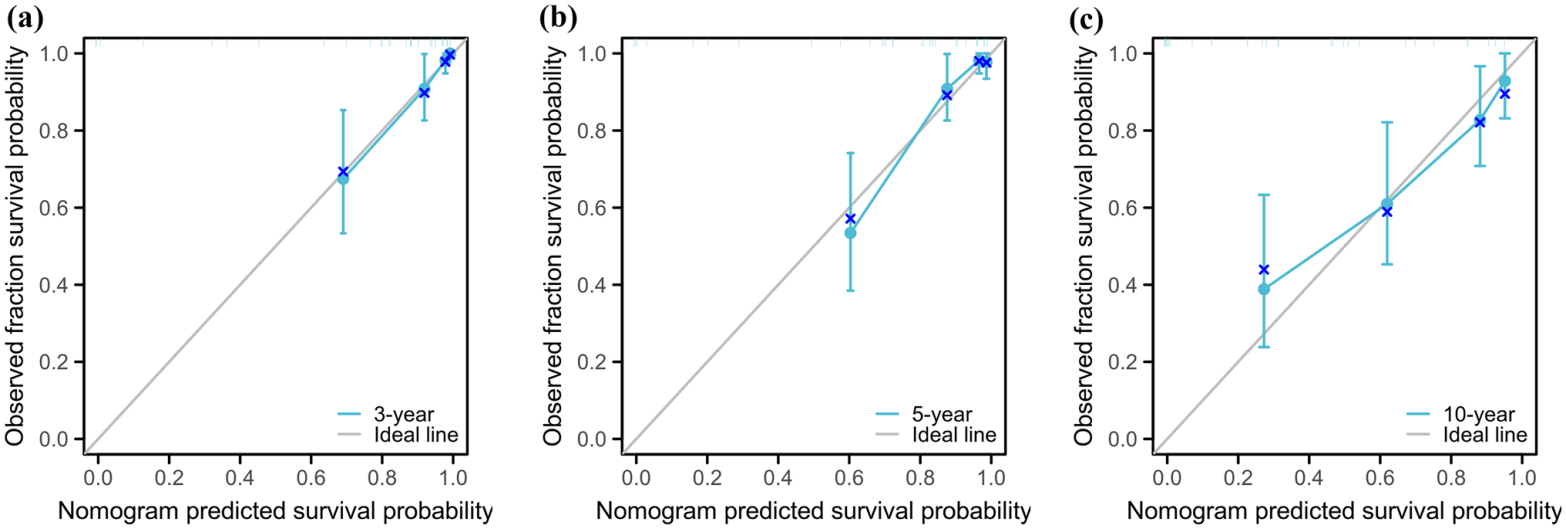
3 year (**a**), 5 year (**b**), and 10 year (**c**) overall survival nomogram internal verification curves

**Fig. 7:**
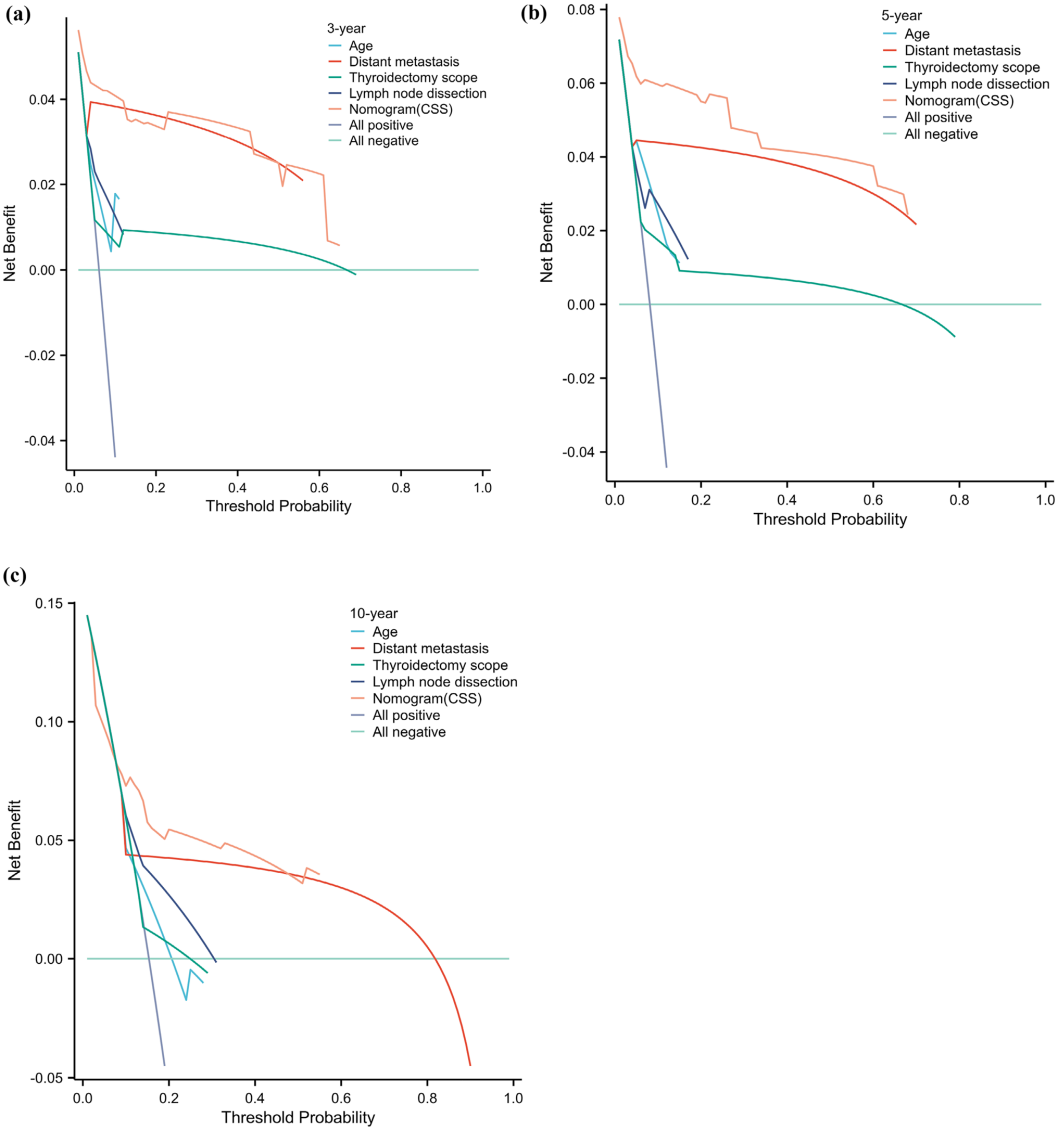
Each independent predictive factor with the established nomogram in the one DCA curve for assessing the performance of predicting 3 year (**a**), 5 year (**b**), and 10 year (**c**) cancer-specific survival

**Fig. 8:**
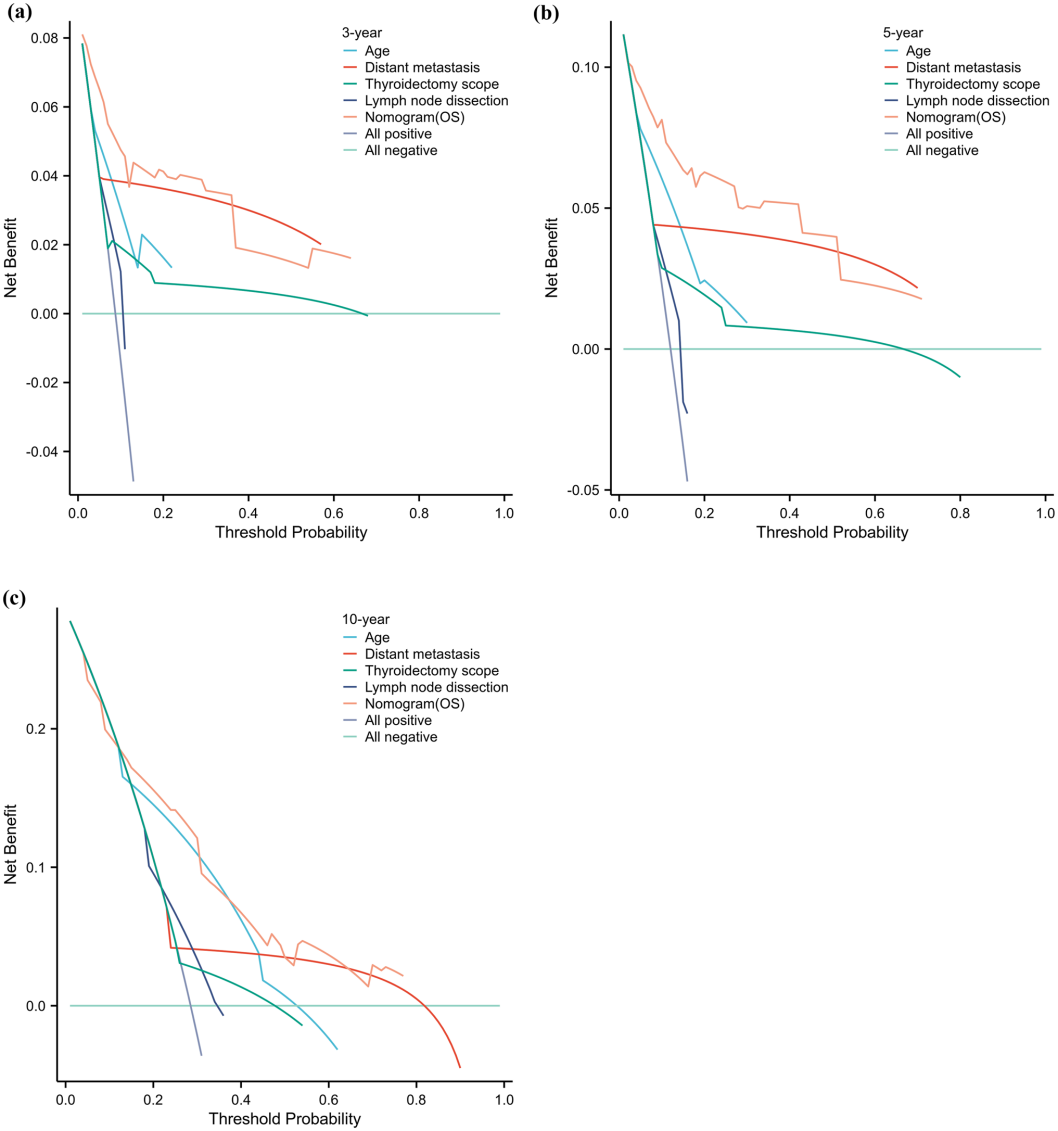
Each independent predictive factor with the established nomogram in the one DCA curve for assessing the performance of predicting 3 year (**a**), 5 year (**b**), and 10 year (**c**) overall survival

**Fig. 9:**
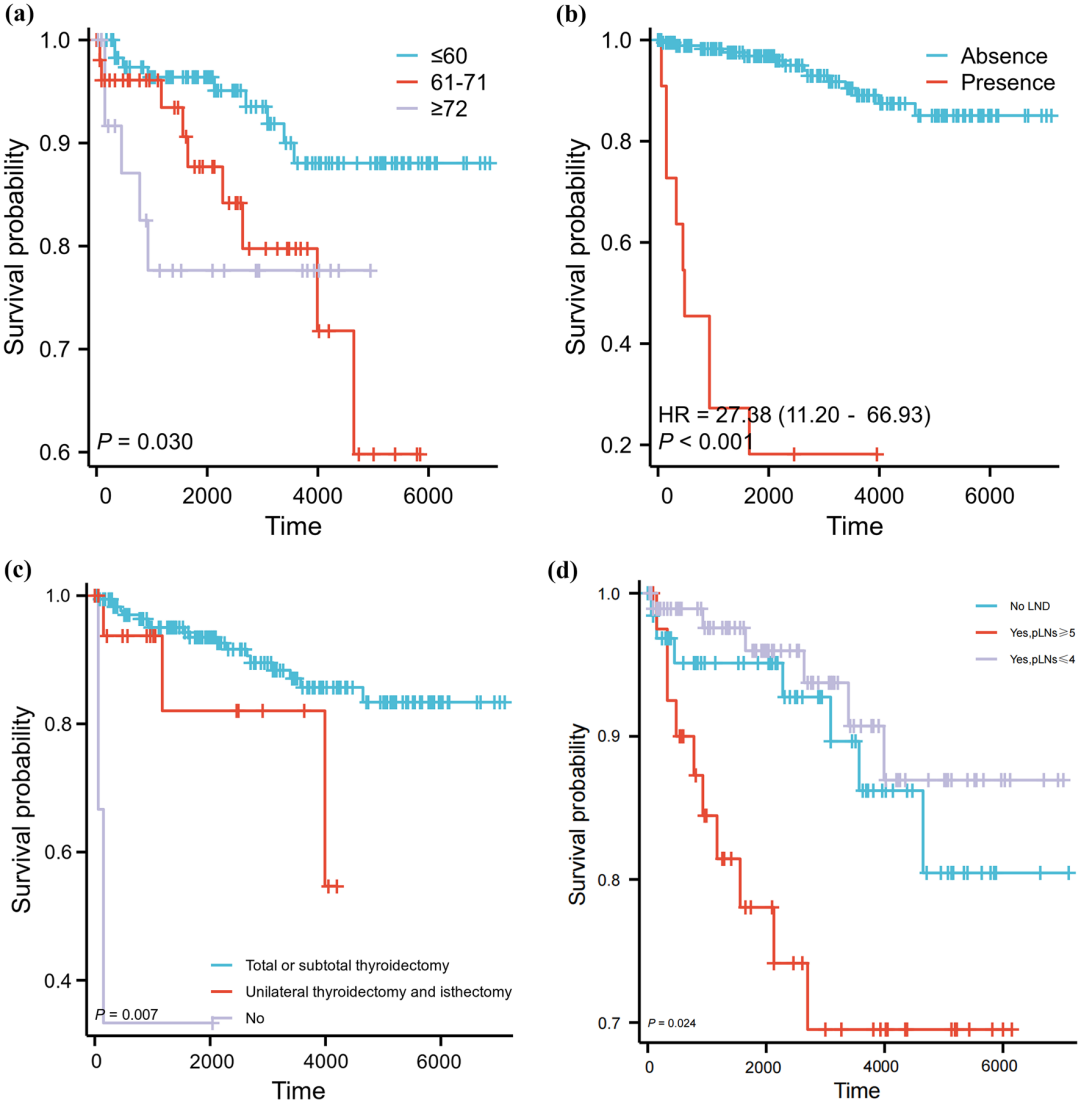
Kaplan–Meier survival curves with respect to CSS were plotted for each significant independent predictor. **a** Age (years), **b** Distant metastasis, **c** Thyroidectomy scope, **d** Lymph node dissection status. Note: Time (days)

**Fig. 10:**
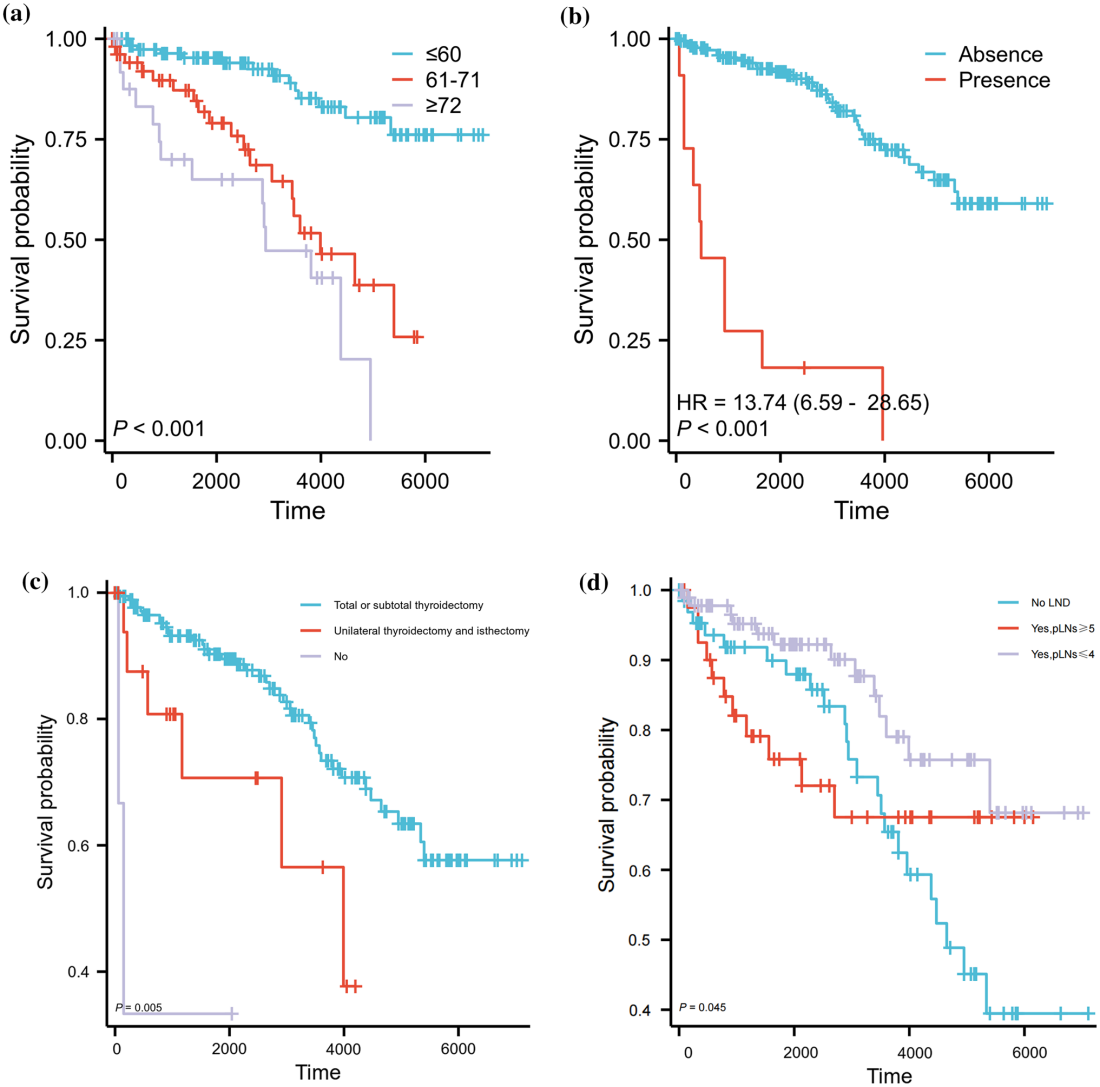
Kaplan–Meier survival curves with respect to OS were plotted for each significant independent predictor. **a** Age (years), **b** Distant metastasis, **c** Thyroidectomy scope, **d** Lymph node dissection status. Note: Time (days)

The C index of the prognostic models for CSS and OS in the MMFCC patient population was 0.838 and 0.794, respectively, and the time-dependent AUC curves (Fig. 4a, b) can show the trends of the AUC indices of the factors and nomograms, thus reflecting the good discriminatory ability of the aforementioned nomograms. In addition, we used a similar self-service resampling program to plot 3-, 5-, and 10-year calibration curves for CSS and OS nomograms for this model (Figs. 5a, b, c, 6a, b, c) and compared each independent predictor in the DCA curves (Figs. 7a, b, c, 8a, b, c) along with the established nomograms, and it can be concluded that the model performs well in terms of predictive accuracy and clinical applicability, and it can be seen that nomograms have superior predictive performance compared to individual factors. We used the Kaplan–Meier method to plot survival curves for CSS and OS for each significant independent predictor (Figs. 9a, b, c, d, 10a, b, c, d).

## Risk stratification based on CSS and OS prognostic nomograms

**Fig. 11:**
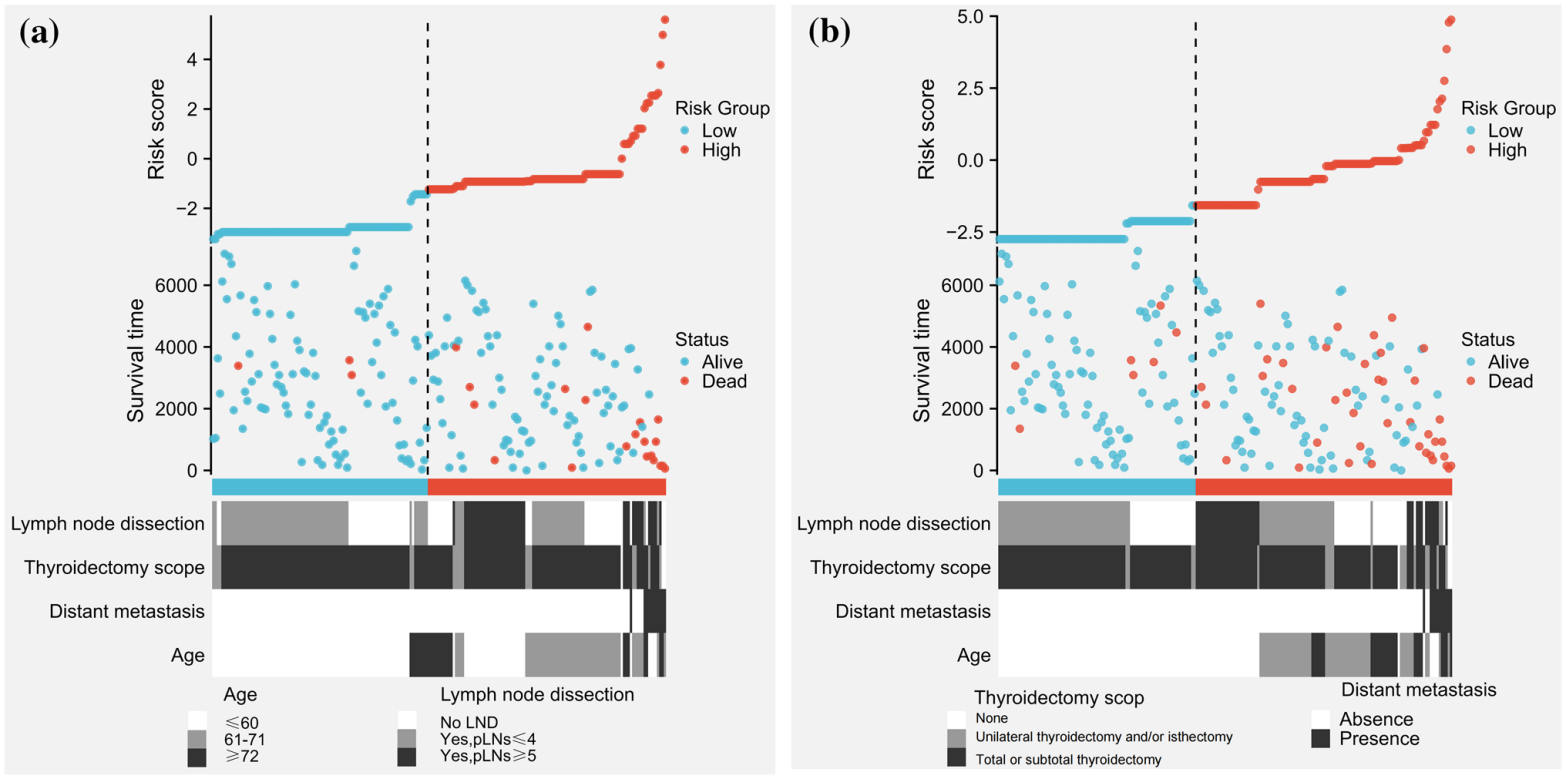
Risk factor maps of the prognostic prediction nomograms for CSS (**a**) and OS (**b**). Note: Survival time (days)

By summing the predictive scores corresponding to the different variables of the CSS and OS prognostic nomograms to calculate the total score, we could predict the survival status of the MMFCC patient population. As shown in Table 4, we determined the cut-off value by calculating the Jordon index, which led to the division into two subgroups, i.e., the low-risk group and the high-risk group. The actual survival rates of the MMFCC patient population in the two groups obtained by the CSS prognostic nomogram with the total score according to this division criterion were 94.5% and 61.1% (*P* < 0.001). According to the OS prognostic prediction nomogram, the actual survival rates of the two groups with this division criterion were 91.3% and 56.5% (*P* < 0.001). The differences in the distribution of the population proportion, survival time, and survival status between the high-risk and low-risk groups can be visualized by plotting the risk factor maps (Fig. 11a, b).

**Table 4:** Risk stratification distribution of 200 patients with mixed medullary and follicular cell carcinoma

		LR	HR	Total	*P* value
CSS	Scoring range	< 72.5	≥ 72.5		
	Dead (attributable to MMFCC)	9 (5.5%)	14 (38.9%)	23	
	Alive or dead of other cause	155 (94.5%)	22 (61.1%)	177	
	Total	164	36		< 0.001
OS	Scoring range	< 63.5	≥ 63.5		
	Dead	10 (8.7%)	37 (43.5%)	47	
	Alive	105 (91.3%)	48 (56.5%)	153	
	Total	115	85		< 0.001

*LR* low-risk, *HR* high-risk, *MMFCC* mixed medullary and follicular cell carcinoma, *CSS* cancer-specific survival, *OS* overall survival

## Discussion

Although MMFCC has long been detected in the clinic, its tumor origin, incidence, prognosis, and clinicopathological features have been inconclusive in the academic community due to its extreme rarity. Papotti et al. (1997) analyzed all thyroid cancer cases over a 20 year period in the Department of Pathology of the Mayo Clinic and the University of Turin and found that MMFCC accounted for a mere 0.15% of all thyroid cancer cases. In the present study, the prevalence of MMFCC was derived from a retrospective analysis of 215,232 patients with pathologically confirmed thyroid malignancies in the SEER database between 2000 and 2020 as 0.10%. The pathogenesis of MMFCC is still unclear, and may be importantly linked to its unique biopathological and molecular etiological properties. The mechanisms currently believed by the academic community to be potentially involved in the development of MMFCC include: (1) multiple different primary tumors occurring consecutively in the same region due to coincidences; (2) alteration of the microenvironment by the preexisting tumors, which increases the likelihood of re-tumorigenesis; (3) related tumors originate from common stem cells and are able to differentiate into mixed tumors of both follicular cell origin and parafollicular C-cell origin; (4) mutations in related genes, such as Ras, BRAF, and RET, lead to tumor dysplasia or chemotaxis; (5) stimulation of oncogenic factors leads to the simultaneous differentiation of different types of tumors; and (6) genetic factors, which have demonstrated that hereditary MTCs are more prone to develop MMFCC compared to sporadic MTCs. Although the above hypotheses need to be further demonstrated, it is believed that the development of MMFCC is the result of a combination of mechanisms (Ryan et al. 2015; Thomas et al. 2021; Vantyghem et al. 2004; Sadow et al. 2010).

Immune diseases such as Hashimoto’s thyroiditis (HT) may be associated with the occurrence and development of PTC (Li et al. 2023; Xu et al. 2021; Chen et al. 2013). It has been reported that basal serum calcitonin levels are significantly higher in HT patients (Karanikas et al. 2004), which may be related to MTC or its precancerous-related diseases (micro-MTC and neoplastic C-cell hyperplasia) (Guyetant et al. 1994). However, MTC with HT is still rare, and the possible relationship between HT and the occurrence and development of MTC and PTC needs to be further explored (Malpani et al. 2020). Zhang et al. (2023) classified papillary thyroid carcinoma (PTC) in combination with MTC, Type I: i.e., MM-PTC, which showed two different histologic types in the same lesion and closely intertwined; Type II: Collision MTC/PTC, which showed two pathologic types in the same lesion; Type III/IV are both two pathologic types located in different lesions, with Type III. Synchronous MTC/PTC in the same glandular lobe and Type IV. Synchronous MTC/PTC not in the same glandular lobe. Before this study, there have been confusing reports of DTC and MTC combined tumors without distinguishing “mix, collision and synchronous” (Wong et al. 2012), which may not have the same histopathological features and prognosis. In our study, we retrospectively analyzed the clinical data of 200 patients with MMFCC.

Our study suggests that there may be a significant difference in development and prognosis between MMFCC and MTC, and that MTC may have a prognosis that is intermediate to the prognosis of between MM-PTC and MM-FTC. Notably, Liu et al. (Liu et al. 2017) compared 60 cases of MSTC with follicular thyroid carcinoma (FTC) and PTC from 2004 to 2013 in the SEER database and found that the prognosis of MSTC was similar to that of PTC and FTC when matched for all influential factors. Nonetheless the prognosis of MMFCC remained unclear, probably because previous studies failed to distinguish MM-PTC from MM-FTC, so the present study subsequently compared the two between groups for relevant factors, but we found that except for tumor size, including CSS and OS, no significant differences were seen in other variables, while the differences in subtype did not show a direct association with prognosis in the Cox regression analysis of CSS and OS in MMFCC patients. In contrast, Sandilos et al. (2023) analyzed patient information from the NCBD database and concluded that MM-FTC had a worse prognosis compared with DTC and MTC, while the OS of MM-PTC was better than that of MTC but worse than that of DTC. Among them, the association between OS of MM-PTC and gender, insurance and income status, tumor size, positive margin status, and DM were significantly associated, while DM and tumor multifocality were independent predictors of OS in MM-FTC patients, but unfortunately this study did not investigate the association of related factors with CSS in MMFCC patients. The present study showed that MM-PTC was more common than MM-FTC in the MMFCC patient population with a percentage of 69.5%, which is similar to the results of previous studies (Negura et al. 2023), and the reason for this may be the higher percentage of PTC possessed in DTC. Similar to the findings of Negura et al. (2023), our study also concluded that age is an important factor in the prognosis of patients with MMFCC, which was not obtained by Sandilos et al. (2023), which may be related to the delineation of inappropriate age cut-offs. In terms of clinicopathological features, although tumor size, ETE status, and LNM region reflected significant differences with patients’ prognosis in univariate Cox regression analyses, the above factors did not appear in the final results, but the strength of MMFCC aggressiveness may be closely related to them (Negura et al. 2023), which can be explored in depth in future studies. The most frequent sites of DM in thyroid cancer are lung, bone, brain, and liver (Shaha et al. 2001), and MMFCC patients who developed DM in this study had shorter survival compared to those who did not, which is the same as in previous studies (Negura et al. 2023; Sandilos et al. 2023).

Due to the multiple pathological features and biological behaviors of MMFCC, most of the patients do not have clinical manifestations, and the difficult preoperative examination may be one of the reasons for the poor prognosis of MMFCC. The diagnostic ability of ultrasound-guided fine needle aspiration biopsy (US-FNAB) is often limited by incomplete sampling, operator’s puncture technique, and specimen preparation and reading process (Zhang et al. 2023), and the diagnosis can only be confirmed by postoperative pathology and immunohistochemistry. However, it is worth noting that preoperative calcitonin and carcinoembryonic antigen (CEA) serology, combined genetic testing of the puncture specimen (e.g., BRAF V600E mutation, RAS mutation, and RET/PET rearrangement, etc.), and ultrasound-guided core needle biopsy, if necessary, can help surgeons to make a more accurate diagnosis in the preoperative period. Moreover, for patients with preoperative diagnosis of DTC confirmed by US-FNAB in combination with elevated serum calcitonin and/or CEA, frozen pathology should be performed intraoperatively to avoid missed diagnosis and thus compromising the scope of surgery.

There is no targeted treatment standard for MMFCC, and some patients have a poor prognosis. Cox regression analysis in this study showed that the thyroidectomy scope was an independent risk factor for the prognosis of MMFCC patients, both in terms of CSS and OS, and the Kaplan–Meier curves can also be visualized to analyze that the prognosis of MMFCC patients who had their glands surgically resected was much better than that of those who had not undergone surgical treatment. Notably, in terms of LND, we found that compared with MMFCC patients without LND, the prognosis of the MMFCC patient group with the number of positive lymph nodes less than or equal to 4 after LND was better, whereas the prognosis of the MMFCC patient group with the number of positive lymph nodes greater than or equal to five was worse, which suggests that high volume LNM has a stronger prognostic predictive role. Although most guidelines do not specify the scope of surgery for MMFCC, radical surgery remains the preferred and feasible treatment option to improve the prognosis of patients with MMFCC except in special circumstances, and whether lateral cervical lymph nodes should be cleared should be judged based on preoperative imaging and calcitonin levels (Wells et al. 2015). For more complicated cases, we recommend the implementation of the multi-disciplinary team (MDT) model, which uses multi-disciplinary collaborative evaluation and discussion, joint diagnosis and treatment, comprehensive assessment of surgical feasibility, surgical modality and timing of surgery, and resection of some of the affected tissues and organs, in order to improve the quality of life in the postoperative period. In addition, the MDT model is also recommended to be applied to postoperative management. When the pathology department makes an accurate judgment of the pathological components of MMFCC, the thyroid surgeon should collaborate with the departments of nuclear medicine, radiotherapy, imaging, and oncology to formulate a plan for the patient’s postoperative treatment and follow-up, in order to detect the recurrence of the tumor and DM as early as possible and to carry out the treatment, so as to improve the patient’s quality of life and prolong the survival time. Although neither radiotherapy nor chemotherapy ultimately became independent predictors of the prognosis of MMFCC patients in this study, due to the diversity and complexity of the pathological features of MMFCC, we believe that the selection of MMFCC treatment regimens and the establishment of follow-up patterns should be adopted as an individualized treatment plan, and that the patients’ postoperative thyroid stimulating hormone (TSH) suppression regimens should be developed based on the stratification of the risk of recurrence of the tumor and the risk of adverse effects of TSH suppression therapy. It has been found that radioisotopes should be performed postoperatively when MMFCC tumor tissue thyroglobulin (+) is present, as it is more effective in the treatment of local residual tumor, LNM foci < 1 cm in diameter, and small DM lesions (Kostoglou-Athanassiou et al. 2004). Zhang et al. concluded that calcitonin and CEA levels affect the prognosis of MMFCC patients to a certain extent, and calcitonin is more sensitive than CEA (Zhang et al. 2023), so we suggest that patients with MMFCC should be routinely rechecked for the tumor markers calcitonin and CEA after surgery. For advanced postoperative patients with rapid progression and symptoms and MMFCC patients who cannot be treated surgically or with DM, external beam radiation therapy, chemotherapy, and targeted therapy can be used, but their efficacy is still controversial (Hadoux et al. 2017; Tuttle et al. 2010).

Nevertheless, due to the rarity and complexity of MMFCC, there is no detailed diagnosis and treatment for MMFCC in the academia, and previous studies have not established a clinical prognostic nomogram for it. In this study, age at diagnosis was derived from the analysis of the demographic, clinicopathological characteristics, and therapeutic survival data of 200 patients with MMFCC in the SEER database from 2000 to 2020, presence of DM, thyroidectomy scope, and LND status were significantly associated with patient prognosis and were independent predictors of CSS and OS in MMFCC patients. By plotting Kaplan–Meier survival curves for each of the above factors, we could visualize the effects of the relevant differences on patients’ CSS and OS. On this basis, we established prognostic nomograms for CSS and OS in the MMFCC patient population, and the survival probabilities of each MMFCC patient at 3, 5, and 10 years can be easily derived by calculating the relevant scores of each variable. In addition, we performed internal validation and performance evaluation of the nomograms. By plotting time-dependent AUC curves, calibration curves, and DCA curves, we showed that the model performed well in terms of differentiation, accuracy, and clinical applicability. In addition, we risk-stratified the prognosis of patients by the scores in the nomograms and suggested that the high-risk patient group is strongly recommended to use the MDT model to develop a personalized treatment plan and to increase the frequency of review and follow-up after surgery in order to improve the prognosis as much as possible. Despite the good predictive performance and clinical applicability of the prognostic model in this study, the study still has some limitations. First, the SEER database did not collect information on serologic tests such as calcitonin and CEA in patients, which may be important for the prognosis of MMFCC. Second, our study was a retrospective study based on the SEER database, so there may be an inherent selection bias. In addition, the information in the SEER database does not contain information about the molecular biology of genes, patients’ family history, and targeted therapies, all of which may affect the prognosis of MMFCC patients. Finally, although MMFCC is rare in the clinic, effective external validation is more illustrative of model performance, and future studies could conduct joint external validation at multiple centers.

## Conclusions

In conclusion, our study showed that there are significant differences between MMFCC and MTC in terms of clinical characteristics. By identifying independent predictors of prognosis in MMFCC patients, nomograms were established to predict CSS and OS at 3, 5, and 10 years. This model demonstrated good predictive performance and clinical applicability. In addition, a risk stratification scheme based on the prognosis of patients with MMFCC was proposed to help surgeons make postoperative treatment and follow-up plans for patients.

## Data Availability

The data that support the findings of this study are available from the Surveillance, Epidemiology, and End Results (SEER) database at http://www.seer.cancer.gov.
